# Genotype‐phenotype correlation in 99 familial adenomatous polyposis patients: A prospective prevention protocol

**DOI:** 10.1002/cam4.2098

**Published:** 2019-03-21

**Authors:** Junea C. de Oliveira, Danilo V. Viana, Cleyton Zanardo, Erika M. M. Santos, André E. de Paula, Edenir I. Palmero, Benedito M. Rossi

**Affiliations:** ^1^ Oncogenetics Department Barretos Cancer Hospital Barretos SP Brazil; ^2^ Biostatistics Department Barretos Cancer Hospital Barretos SP Brazil; ^3^ Cancer Genetics, Oncology Department Sírio Libanes Hospital São Paulo Brazil; ^4^ Center of Molecular Diagnosis Barretos Cancer Hospital Barretos SP Brazil; ^5^ Molecular Oncology Research Center Barretos Cancer Hospital Barretos SP Brazil; ^6^ Barretos School of Health Sciences, Dr. Paulo Prata – FACISB Barretos SP Brazil

**Keywords:** adenomatous polyposis coli, colorectal neoplasms, genotype, germline pathogenic variants, phenotype

## Abstract

**Background:**

Familial adenomatous polyposis (FAP) is a syndrome caused by germline pathogenic variants in the tumor suppressor gene adenomatous polyposis coli (*APC*). Identification of *APC *pathogenic variants sites and the genotype‐phenotype correlation are important for characterizing, monitoring, and treating members of affected families. The aim of this study was to correlate genotype‐phenotype of Brazilian individuals carrying *APC *pathogenic germline variants and that have FAP.

**Methods:**

The polyposis phenotype of 99 individuals from 35 families between July 2013 and December 2014 were prospectively evaluated based on the InSIGHT polyposis staging classification. Seven extra‐colonic manifestations were assessed and the clinical manifestations correlated with the *APC* genotype.

**Results:**

The age of the study participants ranged from 12 to 67 years (median of 29 years). Twenty‐six *APC *pathogenic variants were identified. Fifty‐five cases harbored nonsense pathogenic variants (55.6%). Frameshift alterations were noted in 39 cases (39.4%). Aberrant splicing was noted in 1 case (1%). Rearrangements were observed in 3 cases (3%). An association between nonsense variants and rearrangement was noted in 1 case (1%). The genotype‐phenotype correlation analysis led the identification of classic FAP in 94 cases (94.9%). Profuse polyposis was identified in 5 cases (5.1%). Thirty‐six cases were diagnosed with cancer of which 29 cases (80.6%) were colorectal cancer, 1 case (2.7%) was brain cancer, 4 cases (11.2%) were papillary thyroid cancer, and 2 cases (5.5%) were stomach cancer. The extra‐colonic manifestations included 9 individuals with desmoids tumors, 10 with osteomas, and 9 with congenital hypertrophy of the retinal pigment epithelium.

**Conclusions:**

The genotype‐phenotype correlation in Brazilian individuals with FAP revealed specific findings not previously reported for other cohorts, demonstrating the relevance of knowledge regarding the variable pathogenic variants and clinical presentation in different populations for adequate individual clinical management of patients harboring this medical condition.

## 1 BACKGROUD

Familial adenomatous polyposis (FAP) is a hereditary colorectal cancer (CRC) syndrome caused by pathogenic germline variants of the *adenomatous polyposis coli* (*APC*) tumor suppressor gene located on chromosome 5(5q21‐22;OMIMNM_000038.5). FAP is characterized by the development of hundreds to thousands of adenomatous polyps in the colon and rectum. The global prevalence of FAP is estimated at 3 cases/100,000 people; the prevalence in Brazil is 1 case/30,000 people.^1^, ^2^, ^3^

FAP patients exhibit different phenotypes and the associated extracolonic manifestations vary considerably. The incidence of extracolonic manifestations is as high as 40%. For many decades, CRC was the main cause of death among FAP patients; however, this is changing due to a more thorough understanding of the disease, early diagnosis, and prophylactic surgery. Currently, desmoids tumors and upper gastrointestinal tumors are the main causes of death and therefore these tumors require long‐term monitoring.^4^

In general, all patients with pathogenic germline variants of *APC *develop adenomas by age 35. Progression into CRC on average starts in one or more adenomas at age 39 and 95% of patients are affected by age 50. However, approximately 7% of patients develop CRC soon after the age of 20.^5^

Extracolonic manifestations differ according to the genotype. Upper gastrointestinal lesions, such as duodenal adenomatous polyps occur in up to 90% of cases. Duodenal papillary or periam pullary polyps develop in up to 50% of cases. The risk of an FAP patient developing periam pullary carcinomas and duodenal or papillary carcinoma is increased 100‐fold to 300‐fold compared to that of the overall population. The development of brain tumors in FAP patients is known as Turcot syndrome, while Gardner syndrome is the association of FAP with duodenal periam pullary adenomas, congenital hypertrophy of the retinal pigment epithelium (CHRPE), jaw osteomas, odontomas, desmoid tumors, thyroid tumors, and biliary duct tumors.^5^, ^6^ Desmoid tumors occur in up to 30% of FAP cases and are associated with high morbidity. Risk factors for the occurrence of desmoid tumors include positive family history, being female, previous abdominal surgery, and *APC *pathogenic variants at codon 1309.^7^ FAP patients without a family history may represent cases of de novo variants, which correspond to approximately 20%‐30% of patients.^8^ FAP generally exhibits complete penetrance. According to the literature, these verities of FAP is associated with the genotype‐phenotype correlation of *APC*. Analyses of the *APC *gene‐pathogenic variants status and the phenotype allow for the identification of groups of high‐risk patients which in turn allow for the application of targeted and individualized monitoring and preventative measures.

## 2 METHODS

### 2.1 Study design and ethical standards

The study was a prospective single‐arm, single‐center trial conducted from July 2013 through December 2014. The study design was reviewed and approved by the Barretos Cancer Hospital research ethics committee on 28 June 2013 under registration number 317.264 and was assigned a Certificate of presentation for Ethical Appreciation (CAAE) number 15850813.8.0000.5437. The trial was retrospectively registered in the Brazilian Clinical Trials Registry (ReBEC) on 3 February 2018 (number RBR84qkkv). Written informed consent was obtained from all participants or their guardians.

All index patients and participating relatives who signed the informed consent for the trial participation were analyzed in this study. The authors confirm that all ongoing and related trials for this intervention are registered. All procedures performed in this study were in accordance with the ethical standards of the institutional research committee, and with the 1964 Helsinki declaration and its later amendments. Patients diagnosed with classic FAP and carriers of pathogenic germline variants of *APC *were included. Patients were subjected to directed clinical interviews including personal and family history of CRC and extracolonic manifestations, genogram plotting, physical examination, and genetic counseling prior to and following genetic testing.

### 2.2 Testing protocol

FAP staging was performed based on colonoscopy and anatomic‐pathological examination according to the InSIGHT Polyposis Staging System (IPSS).^9^ The following seven extracolonic manifestations were assessed by specific tests: desmoid tumors of the abdomen by computed tomography (CT); duodenal and gastric lesions (polyps or tumors) by upper gastrointestinal endoscopy (UGIE); thyroid disorders by Doppler ultrasound; brain lesions by magnetic resonance imaging (MRI); osteomas by radiographs of the skull, mandible, and facial bones; and CHRPE by ophthalmoscopy. The ophthalmoscopy was performed by oncologists during consultation and confirmed by an ophthalmologist. Clinical management based on test results was performed in accordance of standard clinical practice a tour site. For patients whose test findings required no specific clinical or surgical intervention, imaging, and endoscopic exams were routinely performed each year. A brain MRI was performed every 3 years for patients with no abnormal findings.

### 2.3 Families and patients

A total of 35 families with pathogenic variants of *APC *were assessed and a total of 99 individuals received follow‐up at Barretos Cancer Hospital (BCH). The genotype‐phenotype correlation was assessed for all participants.

#### 2.3.1 APC gene analysis

The genetic tests were performed at the BCH Center of Molecular Diagnosis using DNA extracted from peripheral blood. All encoding exons and flanking intronic regions were amplified by polymerase chain reaction (PCR) and subjected to conventional bidirectional sequencing (Sanger method) using the ABI3500xL platforms described by Palmero et al.^10^ The primers for amplification were designed and validated by the technical‐scientific staff of the BCH Center of Molecular Diagnosis. Sequencing was performed using a BigDyeTerminatorv3.1Cycle Sequencing Kit (Life Technologies). The electropherograms were analyzed using SeqScape software (Applied Biosystems). All samples with possible deleterious variants were re‐extracted and subjected to a second PCR amplification followed by bidirectional sequencing of the involved region. Family members tested for pathogenic variants previously identified in the index case were included for genetic testing targeting the variant segregated within the family. All identified variants were graded based on their deleterious potential and clinical effect using the Leiden Open Variation Database (LOVD Colon Cancer Gene Variant Databases), Human Genome Variant Database (HGMD), and ClinVar database.

### 2.4 Statistical analysis

The data were subjected to descriptive statistics; analysis was performed using SPSS V19.

## 3 RESULTS

A total of 99 individuals with FAP from 35 families with pathogenic germline variants of *APC *were analyzed. Table 1 describes the families included in the study and the *APC* variants identified. In total, 26 different pathogenic germline variants in *APC *were identified and family #12 exhibited two different pathogenic variants.

**Table 1:** Pathogenic variants detected among the 35 families analyzed in the study and the number of family members

Family	*APC *Mutations	N
1	p.Gln1062*(c.3183_3187delACAAA)	11
2	p.Arg213Ter(c.637C>T)	11
3	p.Glu1309Aspfs*4(c.3927_3931delAAAGA)	5
4	p.Tyr1179Ter(c.3537_3543delTAGTTTA)	1
5	p.Arg302Ter(c.904C>T)	4
6	p.Gln1041Ter(c.3121C>T)	2
7	p.Asp849Glufs*11(c.2547_2550delTAGA)	1
8	p.Ser1104Glufs*20(c.3310_3316delTCACGGG)	2
9	p.Tyr986Ter(c.2958T>G)	13
10	p.Arg283Ter(c.847C>T)	1
11	p.Arg302Ter(c.904C>T)	5
12	p.Glu578Ter(c.1732G>T) Deletion of exon 15	3
13	p.Tyr1166Ter(c.3498T>A)	1
14	p.Asn1017Metfs*4(c.3050_3053delATGA)	1
15	p.Arg805Ter(c.2413C>T)	2
16	p.Gln1062*(c.3183_3187delACAAA)	1
17	Heterozygous deletion of exons 1 to 15	1
18	p.Glu1309Aspfs*4(c.3927_3931delAAAGA)	1
19	p.Arg302Ter(c.904C>T)	2
20	p.Gln1328Ter(c.3982C>T)	1
21	p.Arg283Ter(c.847C>T)	2
22	p.Arg554Ter(c.1660C>T)	1
23	p.Met720Trpfs*7(c.2157delT)	2
24	p.Arg302Ter(c.904C>T)	3
25	p.Gln1062*(c.3183_3187delACAAA)	1
26	p.Glu1309Aspfs*4(c.3927_3931delAAAGA)	2
27	p.Gln1260Tyrfs*6(c.3776_3777dupTA)	4
28	p.Ser713Ter(c.2138C>G)	3
29	p.Gln1062*(c.3183_3187delACAAA)	2
30	p.Arg232Ter(c.694C>T)	1
31	p.Gln541Thrfs*19(c.1620dupA)	4
32	c.1958+3A>G	1
33	p.Arg232Ter(c.694C>T)	2
34	p.Ile1060Ter(c.3178delA)	1
35	p.Glu287Alafs*2(c.856_859dupCATG)	1
Total		99

N, number of individuals.

Table 2 describes the patient staging according to IPSS‐Colon 9. Thirty‐five patients were characterized as stage 4 that included 29 cases of adenocarcinoma and 6 cases of high‐grade carcinoma.

**Table 2:** Staging of all patients according to the InSIGHT Polyposis Staging System for Colon (IPSS‐Colon)^9^

Stage	Polyp Description	n	%
0	< 20 polyps, all<5mm	0	0
1^a^	20‐200 polyps,most <5mm, none >1cm	51	51.5
2^a^	200‐500 polyps, <10 that were >1cm	13	13.1
3^a^	500‐1000 polyps or any number of polyps if 10‐50 were >1cm and amenable to complete polypectomy	0	0
4^a^	>1000 polyps and/or any polyps grown to confluence and not amenable to simple polypectomy, or adenoma with high‐grade dysplasia	35	35.4
	Total	99	100

a Presence of high‐grade dysplasia warrants upstaging of patient to stage 4.

Characteristics of the residual rectal polyps of patients subjected to prophylactic surgery with rectal preservation are described in Table 3. Eighty‐one of the 99 individuals analyzed were subjected to colon surgery for which rectal preservation was attained in 53 of the patients. Two cases categorized as stage 4 exhibited high‐grade dysplasia. These 2 cases were from the same family with variant p.Glu1309Aspfs*4 (c.3927_3931delAAAGA). Another case that had variant p.Ser1104Glufs*19 (c.3310_3316delTCACGGG) exhibited recurrence of rectal adenocarcinoma.

**Table 3:** InSIGHT Polyposis Staging System for the Rectum (IPSS‐Rectum)^9^ staging of 53 patients subjected to colon surgery with rectal preservation

Stage	Polyp Description	n	%
0	0‐10 polyps, all <5mm	0	0
1^a^	10‐25 polyps, most <5mm, none >1cm	50	94.3
2^a^	10‐25 polyps, any >1cm, amenable to complete removal	0	0
3^a^	>25 polyps amenable to complete removal, any incompletely removed sessile polyp, or any evidence of low gastrointestinal bleeding even if incompletely excised	0	0
4	>25 polyps not amenable to complete removal or any incompletely excised sessile polyp exhibiting high‐grade dysplasia; any invasive cancer	3	5.7
	Total	53	100

a Presence of high‐grade dysplasia warrants upstaging of patient to stage 4.

The study cohort included 50 female patients (50.5%), and 49 male patients (49.5%). The patient ages ranged from 12 to 67 years old. The mean age was 30.7 years old and the median age was 29 years old. The classic polyposis phenotype was identified in 94 cases (94.9%); the other 5 cases (5.1%) exhibited a highly aggressive profuse phenotype. Four of the 5 had pathogenic variants in exon 15, and one had a pathogenic variant in exon 7 (Table 4). All 5 cases of highly aggressive profuse phenotype were in male patients.

**Table 4:** Characteristics of FAP patients from different families with the profuse phenotype

Family	Sex	Age	Mutation	Mutation Type	Exon^a^
30	M	47	p.Arg232Ter(c.694C>T)	Nonsense	Exon 7
15	M	38	p.Arg805Ter(c.2413C>T)	Nonsense	Exon 15
1	M	42	p.Gln1062*(c.3183_3187delACAAA)	Frameshift	Exon 15
3	M	38	p.Glu1309Aspfs*4(c.3927_3931delAAAGA)	Frameshift	Exon 15
8	M	47	p.Ser1104Glufs*19(c.3310_3316delTCACGGG)	Frameshift	Exon 15

a high grade dysplasia

Fifty‐five (55.6%) of the pathogenic variants were nonsense and 39 (39.4%) were frameshift. Three (3%) of the variants were rearrangements and 1 (1%) was a splicing error. In 1 of the 35 families, 2 different pathogenic variants were identified, including 1 nonsense and 1 rearrangement that involved a heterozygous deletion of the entire *APC *(1%). These 2 variants were identified in 3 family members. The characteristics of family #12 are detailed in Table 5.

**Table 5:** Characteristics of family #12

ID	Polyposis Phenotype	Age (y)	Mutation	Gastric adenoma	Duodenal adenoma	Duodenal papilla adenoma	Thyroid disorders
Case 1	Classic FAP with low‐grade dysplasia	33	p.Glu578Ter (c.1732G>T)	No	Low‐grade dysplasia	Low‐grade dysplasia	Multinodular goiter
Case 2	Classic FAP with low‐grade dysplasia	34	Deletion exon 15	Low‐grade dysplasia	Low‐grade dysplasia	Low‐grade dysplasia	Multinodular goiter
Case 3	Classic FAP with low‐grade dysplasia	17	Deletion exon 15	No	Low‐grade dysplasia	Low‐grade dysplasia	Normal

The protocol tests did not reveal any evidence of cancer in 63 of the patients (63.6%). Among the 36 (36.4%) patients with cancer, 29 (80.6%) had CRC, 4 (11.2%) had thyroid cancer, 2 (5.5%) had stomach cancer, and 1 (2.7%) had brain cancer. The characteristics of the molecular abnormalities detected in *APC *among the cancer patients are described in Table 6.

**Table 6:** Molecular abnormalities found in *APC *among cancer patients

Cancer Type	Exon 1–15	Exon 5	Exon 7	Exon 8	Exon 12	Exon 13	Exon 15	Intronic	Total
CRC^a^	1	3	1	5	2	1	15	1	29
Brain	0	0	0	0	0	0	1	0	1
Thyroid	0	2	0	0	0	0	2	0	4
Stomach	0	0	0	0	0	0	2	0	2
Total %	1 2.8%	5 13.9%	1 2.8%	5 13.9%	2 5.6%	1 2.8%	20 55.6%	1 2.8%	36 100%

a Colorectal cancer.

Family #9 contributed the largest number of patients analyzed from a single family (n = 13). One male member was diagnosed with a brain tumor. This individual harbored the variant p.Tyr986Ter (c.2958T>G) in exon 15. At age 14 years, he was subjected to craniotomy for resection of a medulloblastoma and placement of a ventricular shunt. Seven years later, he underwent prophylactic colon surgery that included video laparoscopic total colectomy with ileorectal anastomosis. He also exhibited multiple low‐grade duodenal adenomas, which were also located on the papilla. These lesions were treated by serial resections via upper gastrointestinal endoscopy (UGIE).

Four female patients were diagnosed with papillary thyroid carcinoma. Two of the patients were members of family #2 and had the p.Arg213Ter(c.637C>T) variant in exon 5. The other 2 patients were unrelated from families #1 and #29 and each exhibited the same variant, p.Gln1062Ter(c.3183_3187delACAAA) in exon 15.

Stomach cancer without an association with CRC was diagnosed in 2 individuals from different families; each had a pathogenic variant in exon 15. One patient was a 43‐year‐old male from family #9, had the p.Tyr986Ter(c.2958T > G) variant, and also exhibited a mesenteric desmoid tumor. The other patient was a 42‐year‐old female from family #13 who had the p.Tyr1166Ter(c.3498T>A) variant.

We identified 1 case with a de novo pathogenic variant. This patient was a 15‐year‐old female from family #20 with the classic polyposis phenotype and a p.Gln1328Ter(c.3982C>T) variant in exon 15. This patient exhibited low‐grade adenomatous polyps in the colon and rectum, and adenomas with low‐grade dysplasia in the stomach, duodenum, and duodenal papilla, which were removed by serial resection via UGIE.

The genotype‐phenotype correlations for extracolonic manifestations are detailed in Table 7. Nine cases had desmoid tumors: 1 case with a pathogenic variant in exons 5, 7, and 8 and 6 cases with a variant in exon 15. Among the latter, 3 patients were members of family #9 and had the p.Tyr986Ter(c.2958T>G) variant. Ten cases of osteoma were diagnosed. Three of these patients were from family #2 and harbored a p.Arg213Ter(c.637C>T) variant in exon 5. The other 7 osteoma patients were from different families and had pathogenic germline variants in codons 232, 283, 302, 554, 713, 1062, and 1309. Nine cases of CHRPE were diagnosed that included 7 patients from family #1 that had the p.Gln1062*(c.3183_3187delACAAA) variant in exon 15. The eighth case was a patient with a p.Arg805Ter(c.2413C>T) pathogenic variant in exon 15 and the ninth case had a p.Arg232Ter(c.794C>T) variant in exon 7.

**Table 7:** Description of *APC* mutations and correlation with phenotypic abnormalities among the families analyzed

ID	Codon	N	DT	Brain Tumors	Osteomas	CHRPE	Thyroid CN/Ca	Stomach Ca	Duodenal PapillaryCa
32	1958	1	0	0	0	0	0	0	0
17	Deletion of exons 1 to 15	1	0	0	0	0	0	0	0
12	Deletion of exon 15, 578	3	0	0	0	0	0	0	0
23	719	2	0	0	0	0	0	0	0
2	213	11	1	0	3	0	2	0	0
30 33	232	3	1	0	1	1	0	0	0
10 21	283	3	0	0	1	0	0	0	0
5 11 19 24	302	14	1	0	1	0	0	0	0
22	554	1	0	0	1	0	0	0	0
15	805	2	0	0	0	1	0	0	0
14	1017	1	1	0	0	0	0	0	0
7	849	1	1	0	0	0	0	0	0
6	1041	2	1	0	0	0			0
1 16 25 29	1062	15	0	0	1	7	4	0	0
27	1260	4	0	0	0	0	0	0	0
20	1328	1	0	0	0	0	0	0	0
31	541	4	0	0	0	0	1	0	0
3 18 26	1309	8	0	0	1	0	0	0	1
35	287	1	0	0	0	0	0	0	0
34	1060	1	0	0	0	0	1	0	0
8	1104	2	0	0	0	0	0	0	0
28	713	3	0	0	1	0	0	0	0
13	1166	1	0	0	0	0	0	1	0
4	1179	1	0	0	0	0	0	0	0
9	986	13	3	1	0	0	0	1	0
Total		99	9	1	10	9	8	2	1

Ca, cancer; CHRPE, congenital hypertrophy of the retinal pigment epithelium; CN, complex thyroid nodules; DT, desmoid tumor; ID, family identification; N, number of individuals.

## 4 DISCUSSION

The genotype‐phenotype correlations of pathogenic germline variants of the *APC *have been thoroughly studied; however, the available data were primarily derived from Europe, North America, and Asia. Little information for Latin America is available despite the fact that the first pathogenic variant in the *APC *in Brazil was detected in 1994 and published in 1998.^11^

In 2013, Torrezan et al published the first Brazilian retrospective study, which assessed 23 families included in the Registry of Hereditary Cancer, AC Camargo Cancer Center, São Paulo from 1998 to 2011. The authors described 14 pathogenic variants in the *APC *and 6 pathogenic variants in the *MUTYH.* Regarding the former, the pathogenic variants primarily involve exons 4, 8, and 15. The pathogenic variants are correlated with the occurrence of gastric and duodenal polyps, osteomas, epidermoid cysts, and desmoid tumors. The most common extracolonic manifestations are gastric and duodenal polyps, which are noted in 79% of the study families. Osteomas are only identified in cases of profuse FAP. Desmoid tumors are associated with several pathogenic variants, and only 2 cases exhibit pathogenic variants after codon 1444. Thyroid tumors and desmoid tumors are associated with pathogenic variants p.Asn1017Metfs*4(c.3050_3053delATGA) in codon 1017 and correlate with more aggressive extracolonic manifestations.^12^

In 2015, a team at the Institute of Biology, Federal University of Pará, Belém, Brazil published a study describing a correlation between *APC *pathogenic variant and colonic phenotype severity and the occurrence of gastrointestinal tumors among residents in the northern region of Brazil. Fifteen individuals from 5 families with FAP were assessed. All of the analyzed individuals exhibited have the same variant in exon 15 of the *APC *(codon 1309‐ c.3956delC). This variant has been frequently described in Japanese studies but not in Brazilian studies. In the southern and south eastern regions of Brazil, this variant is noted in only 9% of FAP cases.^13^

To the best of our knowledge, this study is the largest Brazilian case series analyzed to date. This study is also the first prospective Brazilian study to investigate the correlation with 7 different phenotypes with their corresponding genotypes. Twenty‐six different pathogenic variants in the *APC* were identified. Consistent with reports in the literature, the largest proportion of our case series involved classic FAP (94.9%). Most of the classical FAP cases corresponded to nonsense and frameshift pathogenic variants and exhibited wide variability in their distribution across exons. The pathogenic variants predominantly involved exons 5, 8, and 15 and 1 case involved a complete deletion of exons 1 through 15.^14^

According to the literature, most cases of FAP with the profuse phenotype exhibit a variant in exon 15. In 60% of the cases, the variant is located between codons 1250 and 1556. The most aggressive phenotypes are seen in carriers of pathogenic variants in codon 1309 and include an earlier appearance of profuse polyposis symptoms, a high risk of CRC at an early age, and the occurrence of associated desmoid tumors. In this study, 5 cases exhibited the profuse FAP phenotype, which involved codon 232 of exon 7 and codons 805, 1062, 1104, and 1309 of exon 15. Concordance was related to the aggressiveness of variant in codon 1309. Three families including 8 individuals in total exhibited variant in codon 1309. Three of these patients exhibited CRC. One of these patients was diagnosed at age 17 and also developed colon cancer, jaw osteoma, “in situ” duodenal papillary cancer, and IPSS‐Rectum stage 4 adenomas. Another individual, a 34‐year‐old patient with IPSS‐Rectum stage 4 adenomas, underwent serial proctosigmoidoscopy for polyp resection. To date, the remainder of this group has not exhibited any other phenotypic manifestation, except for gastric and duodenal adenomatous polyps with low‐grade dysplasia.^14^, ^15^

This study detected a substantially aggressive characteristic involving rectal tumor development in a patient from family #8 with profuse FAP and a variant in exon 15 at codon 1104. This patient underwent surgery with rectal preservation for the treatment of polyposis and colon cancer. Unfortunately, 1 year later, he developed a rectal tumor with peritoneal carcinomatosis and died. The literature describes a correlation for intermediate phenotypes of extracolonic manifestations and less than 1000 colonic polyps with this genetic region.^14^

We consider pathogenic variants in codons 1104 and 1309 as being highly relevant for the early appearance of FAP symptoms, the development of disease at a young age, and an increased risk for CRC. Because of this, rectal preservation should be carefully assessed during prophylactic surgery among carriers of variants in codons 1104 and 1309.

Regarding type 2 brain tumor polyposis (BTP), 1 case of medulloblastoma occurred in a 14‐year‐old patient with low‐grade colonic, duodenal, and papillary adenomas and a variant in exon 15 at codon 986. This finding was consistent with reports in the literature that describe a relationship between this type of tumor and variants between codons 686 and 1217.^16^

In this study, the p.Tyr986Ter(c.2958T>G) variant was associated with the occurrence of gastric cancer and desmoid tumors. Thus, carriers of this pathogenic variants require regular monitoring of the upper gastrointestinal tract by means of UGIE and annual CT screenings for desmoid tumors. Prophylactic surgery of the colon for prevention of desmoid tumors is recommended starting at age 25 or later. Among thyroid tumors, the histological type most frequently described in the literature is papillary thyroid carcinoma (PTC). PTC is more frequent among women, which was also the situation in this study. A relationship has been described between PTC and CHRPE when the pathogenic variants involve codons 463‐1387; however, these disorders are not associated when the pathogenic variants involve codons 1220‐1513.^17^

We detected 4 cases of PTC among females in this study with 2 of the cases being from the same family with a variant in exon 5 at codon 213. The other 2 cases involved unrelated patients that had the same variant in exon 15 at codon 1062, did not exhibit an association with CHRPE, but did exhibit duodenal and papillary adenomas and craniofacial osteomas. Based on these findings, patients with pathogenic variants in exons 5 and 15 of *APC*, specifically the p.Arg213Ter(c.637C>T) variant in codon 213 and the p.Gln1062*(c.3183_3187delACAAA) variant in codon 1062, are recommended to undergo annual thyroid ultrasound and UGIE screening.

The literature regarding gastric and duodenal manifestations is controversial. Ficari et al stress the relevance of codon 1395^18^ while other studies describe abnormalities related to pathogenic variants in codons 564‐1465^19^ Carriers of variants in codon 934 are at high risk for gastric, duodenal, and papillary adenomas and the development of neoplasms.^20^ Bertario et al found gastric and duodenal adenomas in carriers of variants in codons 976‐1067.^18^, ^20^ In this study, we found fundic gland polyps (FGP) and low‐grade gastric adenomas (LGA) in carriers of variants in codons 213‐1309. Specifically, for codons 986 and 1166 we identified 2 cases of gastric cancer without CRC, which was consistent with reports in the literature. High‐grade duodenal and papillary adenomas were diagnosed in carriers of variants in codons 213, 805, and 1602 and adenomas with low‐grade dysplasia were found in carriers with variants in codons 213 to 1309. The largest number of cases involved pathogenic variants in codons 213, 986, 1062, and 1309. These findings were consistent with data from according to Enomoto et al and Bertario et al, especially regarding variants in codon 564 on ward. In this study, we also detected 1 case of “in situ” papillary adenocarcinoma with a pathogenic variant in codon 1309, which is associated with the greater aggressiveness of the disease. Overall, the evidence suggests that variants in exons 5 and 15 predispose individuals to papillary adenoma. Thus, annual UGIE is recommended for carriers of variants in codons 213, 986, 1062, and 1309.^19^, ^20^

In contrast to our results that were consistent with previous reports, our findings from this study regarding desmoid tumors diverge substantially from the literature. According to Caspari et al,^15^ pathogenic variants between codons 1444 and 1580 predispose individuals to desmoid tumors in association with other extracolonic manifestations. Eccles et al identified multiple desmoid tumors among FAP patients with variants in codon 1924.^15^, ^21^ In our study, we diagnosed desmoid tumors in 9 patients, including 1 with variants in exons 5, 7, and 8 and 6 with variants in exon 5. Three of these patients were members of family #9 and had the p.Tyr986Ter(c.2958T>G) variant. In contrast to the previously reported pathogenic variants, the pathogenic variants in our cohort involved codons 213, 232, 302, 849, 986, 1017, and 1041.

According to the literature, CHRPE occurs in 70%‐75% of FAP patients and osteomas affect 70%‐90% of patients. These manifestations have been reported in several studies and are associated with variants in different codons. This variation does not allow for the determination of the level of risk or of genotype‐phenotype correlations. These features are associated with variants in codons 767‐1513 according to Bisgaad et al,^22^ 542 to 1309 according to Bertario et al,^20^ and 564 to 1465 according to Enomoto et al.^14^, ^19^, ^23^ In this study, CHRPE occurred in carriers of variants in 3 different codons, 232, 805, and 1062 and osteomas occurred in carriers of pathogenic variants in codons 213, 232, 283, 302, 554, 713, 1062, and 1309. Thus, our current findings for CHRPE and osteomas were consistent with the literature.

Most of the correlations identified in this study were similar to those reported in the literature, but a few atypical cases were noted. Some pathogenic variants require stricter patient monitoring, such as variants involving codons 232, 805, 986, 1062, 1104, 1166, and 1309 since they were related to CRC and associated tumors such as gastric cancer, brain cancer, and desmoid tumors. For the testing protocol used in this study, patients not subjected to prophylactic surgery should undergo annual colonoscopy with resection of the largest polyps in combination with annual UGIE and CT of the abdomen. MRI of the brain is recommended every 3 years. Pathogenic variants in codons 213 and 1062 increased the odds for thyroid cancer, especially for females. Therefore, annual Doppler ultrasound is recommended for these patients. Pathogenic variants in codons 213, 1062, and 1309 predisposed individuals to duodenal and papillary disease. Thus, monitoring should be stricter for patients with these pathogenic variants. For carriers of pathogenic variants in codons, 213, 302, 849, 986, 1017, and 1041, prophylactic surgery is recommended starting at age 25 when possible for the prevention against abdominal desmoid tumors.

## 5 CONCLUSION

Among Brazilian patients with FAP, the genotype‐phenotype correlation exhibited specific characteristics not reported for other cohorts. This fact underscores the importance of a thorough knowledge for specific populations regarding pathogenic variants in *APC *together with the clinical presentation of disease. The application of this type of information in the planning and defining of management strategies for preventive testing might allow for more adequate clinical outcomes.

## References

[1] Petersen GM , Slack J , Nakamura Y . Screening guidelines and premorbid diagnosis of familial adenomatous polyposis using linkage. Gastroenterology. 1991;100(6):1658‐1664. 1673441 10.1016/0016-5085(91)90666-9

[2] Campos F . Adenomatosa Familiar: bases do diagnóstico, tratamento e vigilância. São Caetano do Sul: Yendis Editora Ltda; 2010:424.

[3] NCBI . Transcript Details: NM_000038 [Internet]. 2013 https://portals.broadinstitute.org/gpp/public/trans/details?trans-Name=NM_000038.5. Accessed November 11, 2018.

[4] Jasperson KW . Genetic testing by cancer site: colon (polyposis syndromes). Cancer J. 2012;18(4):328‐333. 22846733 10.1097/PPO.0b013e3182609300

[5] Gallagher MC , Phillips R , Bulow S . Surveillance and management of upper gastrointestinal disease in Familial Adenomatous Polyposis. Fam Cancer. 2006;5(3):263‐273. 16998672 10.1007/s10689-005-5668-0

[6] Houlston R , Crabtree M , Phillips R , Crabtree M , Tomlinson I . Explaining differences in the severity of familial adenomatous polyposis and the search for modifier genes. Gut. 2001;48(1):1‐5. 11115811 10.1136/gut.48.1.1 PMC1728176

[7] Nieuwenhuis MH , Mathus‐Vliegen EM , Baeten CG , et al. Evaluation of management of desmoid tumours associated with familial adenomatous polyposis in Dutch patients. Br J Cancer. 2011;104(1):37‐42. 21063417 10.1038/sj.bjc.6605997 PMC3039799

[8] Rustin RB , Jagelman DG , McGannon E , Fazio VW , Lavery IC , Weakley FL . Spontaneous mutation in familial adenomatous polyposis. Dis Colon Rectum. 1990;33(1):52‐55. 2153067 10.1007/BF02053203

[9] Lynch PM , Morris JS , Wen S , et al. A proposed staging system and stage‐specific interventions for familial adenomatous polyposis. Gastrointest Endosc. 2016;84(1):115‐125.e4. 26769407 10.1016/j.gie.2015.12.029 PMC5570515

[10] Palmero EI , Galvão H , Fernandes GC , et al. Oncogenetics service and the Brazilian public health system: The experience of a reference cancer hospital. Genet Mol Biol. 2016;39(2):168‐177. 27192127 10.1590/1678-4685-GMB-2014-0364 PMC4910553

[11] Rossi BM , Phino PMdSL , Nakagawa WT , Johnson L , Lopes A . Tumores colorretais hereditários. Revista do Colégio. Brasileiro de Cirurgiões. 1998;25(4):271‐280. http://www.scielo.br/scielo.php?script=sci_arttext&pxml:id=S0100-69911998000400010&lng=en.

[12] Torrezan GT , Da Silva F , Santos ÉMM , et al. Mutational spectrum of the APC and MUTYH genes and genotype‐phenotype correlations in Brazilian FAP, AFAP, and MAP patients. Orphanet J Rare Dis. 2013;8(1):54. 23561487 10.1186/1750-1172-8-54 PMC3623842

[13] Moreira‐Nunes CA , Di Felipe Ávila Alcântara D , Lima SF , et al. Presence of c.3956delC mutation in familial adenomatous polyposis patients from, Brazil. World J Gastroenterol. 2015;21(31):9413‐9419. 26309368 10.3748/wjg.v21.i31.9413 PMC4541394

[14] Nieuwenhuis MH , Vasen H . Correlations between mutation site in APC and phenotype of familial adenomatous polyposis (FAP): a review of the literature. Crit Rev Oncol Hematol. 2007;61(2):153‐161. 17064931 10.1016/j.critrevonc.2006.07.004

[15] Caspari R , Olschwang S , Friedl W , et al. Familial adenomatous polyposis: Desmoid tumours and lack of ophthalmic lesions (CHRPE) associated with APC mutations beyond codon 1444. Hum Mol Genet. 1995;4(3):337‐340. 7795585 10.1093/hmg/4.3.337

[16] Hamilton SR , Liu B , Parsons RE , et al. The molecular basis of turcot's syndrome s. N Engl J Med. 1995;332(13):839‐847. 7661930 10.1056/NEJM199503303321302

[17] Cetta F , Montalto G , Gori M , Curia MC , Cama A , Olschwang S . Germline mutations of the APC gene in patients with familial adenomatous polyposis‐associated thyroid carcinoma: results from a European Cooperative Study. J Clin Endocrinol Metab. 2000;85(1):286‐292. 10634400 10.1210/jcem.85.1.6254

[18] Ficari F , Cama A , Valanzano R , et al. APC gene mutations and colorectal adenomatosis in familial adenomatous polyposis. Br J Cancer. 2000;82(2):348‐353. 10646887 10.1054/bjoc.1999.0925 PMC2363293

[19] Enomoto M , Konishi M , Iwama T , Utsunomiya J , Sugihara KI , Miyaki M . The relationship between frequencies of extracolonic manifestations and the position of APC germline mutation in patients with familial adenomatous polyposis. Jpn J Clin Oncol. 2000;30(2):82‐88. 10768871 10.1093/jjco/hyd017

[20] Bertario L , Russo A , Sala P , et al. Multiple approach to the exploration of genotype‐phenotype correlations in familial adenomatous polyposis. J Clin Oncol. 2003;21(9):1698‐1707. 12721244 10.1200/JCO.2003.09.118

[21] Eccles DM , van der Luijt R , Breukel C , et al. Hereditary desmoid disease due to a frameshift mutation at codon 1924 of the APC gene. Am J Hum Genet. 1996;59(6):1193‐1201. 8940264 PMC1914868

[22] Bisgaard ML , Bulow S . Familial adenomatous polyposis (FAP): genotype correlation to FAP phenotype with osteomas and sebaceous cysts. Am J Med Genet Part A. 2006;140(3):200‐204. 16411234 10.1002/ajmg.a.31010

[23] Olschwang S , Tiret A , Laurent‐Puig P , Muleris M , Parc R , Thomas G . Restriction of ocular fundus lesions to a specific subgroup of APC mutations in adenomatous polyposis coli patients. Cell. 1993;75(5):959‐968. 8252631 10.1016/0092-8674(93)90539-3

